# The mixed legacy of the rat estrous cycle

**DOI:** 10.1186/s13293-023-00542-7

**Published:** 2023-09-04

**Authors:** Irving Zucker

**Affiliations:** 1 Department of Psychology, University of California, Berkeley, 2121 Berkeley Way West, Berkeley, CA 94720 USA; 2 Department of Integrative Biology, University of California, Berkeley, 3040 VLSB, Berkeley, CA 94720 USA

**Keywords:** Sex differences, Estrous cycle, Sex bias, Trait variability

## Abstract

**Background:**

The rat estrous cycle first characterized by Long and Evans in 1922 profoundly affected the course of endocrine research. Investigators took advantage of sex steroid hormone fluctuations associated with the cycle to assess hormonal influences on anxiety, depression, food intake, stress, brain structure and other traits. Similarities of the rat estrous and human menstrual cycles facilitated understanding of human reproductive physiology. I assessed the impact of awareness of the estrous cycle on the emergence of a sex bias that excluded female rats from biomedical research.

**Methods:**

Beginning with the 1918 volume of the *American Journal of Physiology* and ending in 1976 when the journal subdivided into several separate disciplinary journals, all studies conducted on rats were downloaded; the use of females, males, both sexes and sex left unspecified was tabulated for 485 articles. A second analysis tracked the number of rat estrous cycle studies across all disciplines listed in PubMed from 1950 to 2021.

**Results:**

The description and awareness of variability associated with the rat estrous cycle was correlated with a precipitous decline in investigations that incorporated both sexes, a marked increase in male-only studies and a striking sex bias that excluded female rats. The number of rat estrous cycles studies increased markedly from earlier decades to a peak in 2021.

**Conclusions:**

The initial description the rat estrous cycle was correlated with a substantial decline in investigations that incorporated both sexes; one result was a marked increase in male-only studies and a striking sex bias that excluded female rats from biomedical research. Recognition of the advantages of studies that incorporate the rat estrous cycle has resulted in recent years in an increase of such investigations. Female rats and females of several other species are not more variable than their male counterparts across traits, arguing for female inclusion without requiring cycle monitoring. There, remain, however, many advantages of incorporating the estrous cycle in contemporary research.

## Highlights

The neglect of females in biomedical research has been well documented in the past decade. The initial description of the rat estrous cycle in 1922 was correlated with a subsequent decrease in studies that utilized female rats, a marked increase in male-only studies, and a sex bias against females based on the erroneous belief that they are more variable than males. Recently there has been a substantial increase in studies that incorporate the rat estrous cycle.

## Introduction

Long and Evans in 1922 [1] first established that the rat estrous cycle consisted of four distinct phases, subsequently labeled proestrus, estrus, metestrus and diestrus, based on vaginal cytology; the cycle recurred every 4 days. They concluded “We now have in our hands for the first time an accurate method for the detection of ovarian function in experimental animals.” Wang in 1923 [2], employing running wheels, reported that the modal locomotor activity cycle of the female rat was 4 days: the peak in wheel-running corresponded to the presence of vaginal cornified epithelial cells indicative of estrus, establishing a close link between the vaginal and behavioral cycles. Slonaker in 1924 [3] also monitoring locomotor activity, established that the 4-day cycle was not manifested prior to pubescence nor after menopause; males did not show any rhythmic fluctuations in activity. These findings alerted experimenters to programmed changes in female rats, absent in males, that might confound analyses of non-reproductive traits and unfortunately, promoted using only males in most studies. In the modern era reports that the free running rhythm of locomotor activity of female rats is much more variable than that of males [4] may have convinced some investigators that female rats are to be avoided.

## Methods

The present analysis tracked how female and male rats were utilized in the years preceding and after the 1922 documentation of the estrous cycle. Rats were chosen as the species of interest because they represented the dominant rodent model prior to the more recent ascendance of mice. The analysis was restricted to the *American Journal of Physiology (AJP)*, because it published articles from a broad array of disciplines and was in operation continuously from 1918 to 1976, at which time it subdivided into multiple separate journals (e.g., *AJP cell physiology*, *AJP heart and circulatory physiology, AJP regulatory and integrative physiology*, etc.). To identify these studies, every report published in *AJP* during the aforementioned years was examined for relevance. Reports using rats were classified as: (1) using both sexes, (2) using only males, (3) using only females, or (4) not specifying sex. A second survey in PubMed tracked the number of studies across all disciplines that monitored the rat estrous cycle beginning in 1950 and ending in 2021 using the tool for automated yearly statistics at PubMed URL: http://dan.corlan.net/medline-trend.html.

## Results

**Fig. 1:**
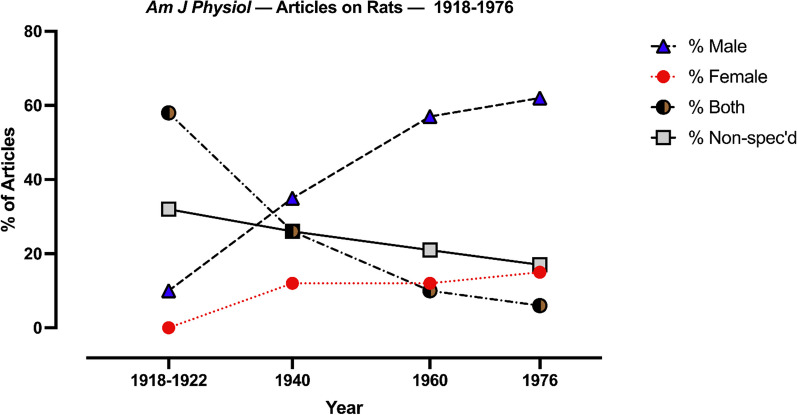
Percent of rat studies across years that utilized males only, females only, both sexes and sex non-specified

Prior to 1922 relatively few rat studies appeared in the *AJP’s* pages; most investigations were conducted on dogs, cats and rabbits descending in number in that order. Beery and Zucker [5] similarly found for the *Journal of Physiology (London)* and the *Journal of Pharmacology and Experimental Therapeutics*, fewer than 10% of animal studies in the first two decades of the twentieth century employed rats and mice. In the years before characterization of the estrous cycle, the majority of rat studies employed both sexes (58%), with very few investigations conducted exclusively on males or females, but fewer studies (*n* = 48) were available than in subsequent years (Fig. 1); many reports failed to specify subject sex. By 1940 (*n* = 57), the study of both sexes had declined from 58 to 26% with further decreases in 1960 (*n* = 181; 10%) and 1976 (*n* = 199; 6%). Contemporaneously, the percent of male-only studies increased from 10% in 1918–22 to approximately 60% in 1960 and 1976. Female-only studies never exceeded 15% in any epoch; the ratio of male to female studies was 4:1.

**Fig. 2:**
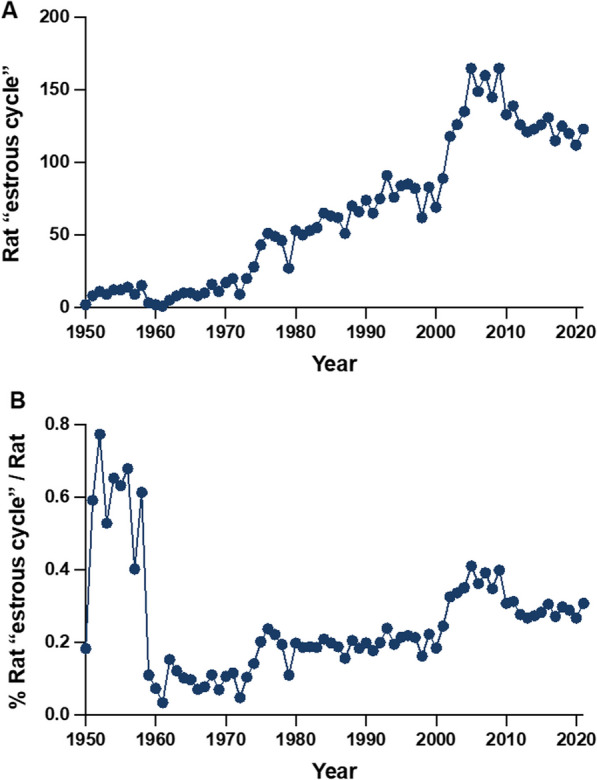
**A** Annual number of published reports on rat estrous cycles as indexed by PubMed from 1950 to 2021. **B** Ratio of rat estrous cycle studies relative to total number of rat studies from 1950 to 2021

Figure 2A plots the number of estrous cycle studies each year beginning in 1950 (the first year the data are available and ending in 2021. There is a sustained increase beginning in the 1970s with a more marked increase beginning in the early 2000s. The ratio of estrous cycle studies relative to the total number of rat studies is elevated in the 1950s, increases in the 1970s and larger increases in the 2000s.

## Discussion

The estrous cycles of several rodent species, first characterized circa 1920, contributed to the subsequent relegation of female rodents to second-class status. Exclusion of female rats by the biomedical community was linked to a hoped-for reduction in trait variance by relying on males, but this did not materialize [6]; many modern studies in several species, including rats [7], mice [8–11] and humans [12, 13] did not detect greater variance in females than males and failed to support the exclusion of females on grounds of increased variability [14].

The markedly lower total number of rat studies in the 1950s (Fig. 2A) elevated the ratio relative to all rat studies (Fig. 2B) but in absolute terms the number of estrous cycle studies during that interval is swamped by the increased numbers beginning in 1980 and charts the substantial progress since 2000 (Fig. 2A), as also reflected in the ratio measure (Fig. 2B) presumably reflecting NIH mandates for including SABV (sex as a biological variable).

A striking sex bias against female rodents, including rats, has been extensively documented in recent years [5, 7, 8, 15, 16]. Prejudice against females was also sometimes evident prior to 1922, as in Hatai and Hammet [17] who wrote “Female rats are not suitable subjects for general studies…in as much as the act of menstruation sets up such changes in the intestinal segment as to cause it to respond in a manner analogous to the segment from young excited male rats.” There have been some recent positive developments incorporating sex as a biological variable. The 2011 paper by Beery and Zucker [5] reported that for 2009, the exclusion of females from neuroscience studies appeared to be the most profound of all biomedical sciences. Woitowich et al. [16] tracked the changes between 2009 and 2019 and reported that over the course of 10 years the largest increases in sex-inclusive studies were seen in neuroscience (29% vs. 63%), and immunology (16% vs. 46%) with smaller increases in endocrinology, general biology, and physiology.

The rat estrous cycle is particularly useful for investigations of behavior, with advantages over the dominant paradigm in which ovariectomized rats are administered hormones or drugs—a time-honored paradigm, but one that does not replicate the timing (e.g., pulsatile secretion), or the concentrations of hormones at different times of day, characteristic of the normal estrous cycle and is better suited to establishing pharmacological than physiological relations.

Investigators have taken advantage of the estrous cycle to investigate the influence of endogenous variations in estrogens and progestins on depression [18], anxiety [19, 20], pharmacology [21, 22], seizures [23], fear [24], hoarding [25], addiction [26], learning [27], aggression [28], cognition [29], metabolism [30], memory [31], body weight [32], food intake [33], sleep [34], circadian rhythms [35], stress [36], brain structure [37] and gene expression [38, 39]. Rocks et al. [40] persuasively argue for “bringing back” the estrous cycle to enhance the resolution and quality of preclinical research and advance the health of women; in this they echo earlier recommendations [41]. It is encouraging that there has been a substantial increase in such studies in recent years (Fig. 2A).

*Perspectives and significance* The estrous cycle has a mixed legacy, facilitating physiologically relevant hormone research on the one hand, and discouraging inclusion of female rats in biomedical research on the other. Many studies can incorporate female rodents without requiring staging of the estrous cycle without increasing variability compared to males.

## Data Availability

All supporting data are available within the article. The datasets used and/or analyzed during the current study are available from the corresponding author on reasonable request.
